# Mapping the future for coral reefs

**DOI:** 10.7554/eLife.72978

**Published:** 2021-09-22

**Authors:** Line K Bay, Emily J Howells

**Affiliations:** 1 Reef Recovery, Restoration and Adaptation, Australian Institute of Marine Science Townsville Australia; 2 National Marine Science Centre, Southern Cross University Coffs Harbour Australia

**Keywords:** *Acropora hyacinthus*, coral reefs, fitness tradeoffs, local adaptation, coral bleaching, heat tolerance, Other

## Abstract

The ability of corals to adapt to global warming may involve trade-offs among the traits that influence their success as the foundational species of coral reefs.

**Related research article** Cornwell B, Armstrong K, Walker NS, Lippert M, Nestor V, Golbuu Y, Palumbi SR. 2021. Widespread variation in heat tolerance and symbiont load are associated with growth tradeoffs in the coral *Acropora hyacinthus* in Palau. *eLife* **10**:e64790. doi: 10.7554/eLife.64790

Climate change has long been recognised as the most serious threat to coral reefs, with heat-induced bleaching being a major cause of reductions in both the total area of living corals and their diversity globally. The future of reefs hinges on multiple factors, such as improved efforts to reduce global warming, the continuation of local management actions to support the health of corals, and the adaptation of both populations and species to climate change (Anthony et al., 2020; Donovan et al., 2021; Knowlton et al., 2021). However, our understanding of the adaptive capacity of corals is relatively poor, which limits our ability to manage and protect existing coral reefs.

Now, in eLife, Steven Palumbi and colleagues at the Hopkins Marine Station of Stanford University and the Palau International Coral Reef Center – including Brendan Cornwell as first author – report new results on the heat tolerance of a key coral species, *Acropora hyacinthus*, in the archipelago of Palau in the western Pacific Ocean (Cornwell et al., 2021). The researchers used a rapid heat-stress assay to evaluate the resistance of *A. hyacinthus* to coral bleaching, and to generate spatial maps of heat tolerance in 221 colonies across 37 reefs in the archipelago.

The good news is that individual heat-tolerant corals can be found in all populations of this iconic species across Palau, and most abundantly in warmer reef microhabitats. This indicates that physiological adaptation to local temperature regimes can occur across short distances (i.e., to warmer habitats within reefs). This is important because these corals can naturally reseed damaged reefs and spread gene variants that are potentially beneficial for adaptation.

Corals live in symbiosis with a diverse range of microbes, and the influences of dinoflagellate algae on both their growth and heat tolerance are well documented (Cunning and Baker, 2013). Cornwell et al. found that corals with fewer symbionts bleached less than those with higher symbiont loads, but these corals also had lower growth rates. While heat tolerance is clearly key to surviving heat-induced bleaching, the ability to grow quickly and reproduce also affects the recovery of a reef (Ortiz et al., 2018). Further work is needed to better understand these trade-offs and their causes in detail. A raft of high-resolution analytical tools – such as functional genomics, metabolomics and proteomics – will help us to understand how symbiotic algae (at the level of genera, species and populations) contribute to the fitness of corals.

The evolutionary outcomes of these trade-offs will depend on their genetic basis. Coral traits, such as heat tolerance and growth, are likely influenced by many genes in both coral and symbiont genomes, each of which may have only a small effect on its own (Fuller et al., 2020). However, interactions between these genes and traits could affect the rate at which corals adapt to change. For instance, adaptation to warming may occur faster than expected if genetic correlations between heat tolerance and growth rate are positive, because selection on either trait has a reinforcing effect on both (Wright et al., 2019). Deploying a combination of different techniques – such as high-throughput phenotyping, genomic analysis of corals and symbionts, and selective breeding – should reveal new insights into the ability of corals to adapt to climate change (Cornwell et al., 2021; Fuller et al., 2020; Howells et al., 2021).

Coral reefs are natural wonders of the world, and their spectacular beauty and biodiversity bring immense social, cultural and economic value to millions of people. But reefs may soon be lost without action to halt their decline and to find ways to support increased heat resilience, both at the local and global level (Knowlton et al., 2021). It is thought that coral reef populations harbouring individual corals that can tolerate heat (and other stressors, such as ocean acidification) can strengthen more vulnerable populations and may therefore be used in restoration and adaptation projects. This means that knowing the distribution of heat-resistant corals across reef systems – and understanding any trade-offs that exist between heat tolerance and growth or reproduction – can inform new management approaches, such as moving heat-resilient corals to reefs that are under threat (Figure 1; van Oppen et al., 2015).

**Figure 1.**
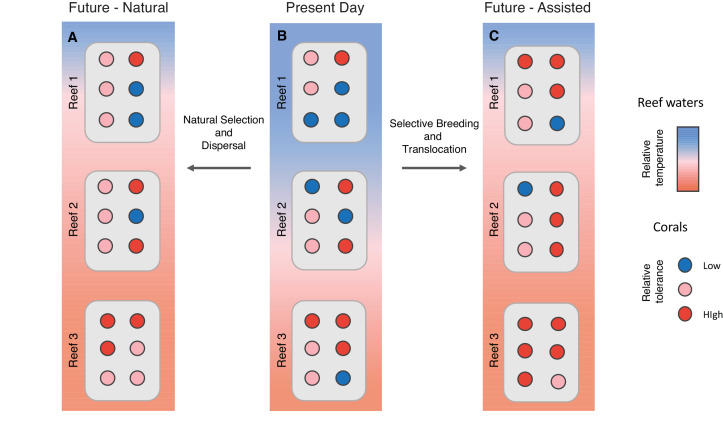
Managing the response of coral reefs to climate change. Knowledge of the distribution and abundance of temperature tolerant corals at present (**A**) can be used to support natural adaptation (**B**) and assisted adaptation (**C**). In this schematic figure, the same three reefs (represented by a rectangular box) exist across a temperature gradient from cool (blue) to warm (red). Each reef contains six colonies of corals (circles); the heat tolerance of each colony can be high (red), intermediate (pink), or low (blue). Under present-day conditions (**A**), warmer reefs contain a higher number of heat tolerant corals than cooler reefs due to local adaptation and other mechanisms. Under future warming (**B**) marine protected areas may conserve sources of corals that evolve through natural selection and spread via dispersal. Assisting these processes (**C**) using management actions, such as selective breeding and translocation, has been proposed to speed up the adaptation rate on some reefs.

A better understanding of the science behind coral adaptation, combined with proper consideration of cultural, social and economic values, is needed to manage the response of coral reefs to climate change.
